# Rapid Detection of VanA/B-Producing Vancomycin-Resistant Enterococci Using Lateral Flow Immunoassay

**DOI:** 10.3390/diagnostics11101805

**Published:** 2021-09-29

**Authors:** Saoussen Oueslati, Camille Gonzalez, Hervé Volland, Vincent Cattoir, Sandrine Bernabeu, Delphine Girlich, Duncan Dulac, Marc Plaisance, Laure Boutigny, Laurent Dortet, Stéphanie Simon, Thierry Naas

**Affiliations:** 1 Bacteriology-Hygiene Unit, Assistance Publique/Hôpitaux de Paris, Service de Bactériologie-Hygiène, Bicêtre Hospital, 94270 Le Kremlin-Bicêtre, France; oueslati.saoussen@gmail.com (S.O.); gonzalezcamille0405@gmail.com (C.G.); sandrine.bernabeu@aphp.fr (S.B.); dgirlich@yahoo.fr (D.G.); laurent.dortet@aphp.fr (L.D.); 2 Team ReSIST, INSERM U1184, School of Medicine Université Paris-Saclay, LabEx LERMIT, 78 Rue du Général Leclerc, 94270 Le Kremlin-Bicêtre, France; 3 Département Médicaments et Technologies Pour la Santé, Université Paris-Saclay, CEA, INRAE, 91191 Gif-sur-Yvette, France; duncan.dulac@cea.fr (D.D.); marc.plaisance@cea.fr (M.P.); stephanie.simon@cea.fr (S.S.); 4 Department of Clinical Microbiology and French National Reference Center for Antibiotic Resistance (Lab Enterococci), Rennes University Hospital, 35033 Rennes, France; vincent.cattoir@chu-rennes.fr; 5 Research and Development Department, NG Biotech, 35480 Guipry, France; laure.boutigny@ngbiotech.com

**Keywords:** LFIA, VRE, vancomycin resistance, rapid diagnostics, immunochromatography

## Abstract

Vancomycin-resistant enterococci (VREs) have become one of the most important nosocomial pathogens worldwide, associated with increased treatment costs, prolonged hospital stays and high mortality. Rapid detection is crucial to reduce their spread and prevent infections and outbreaks. The lateral flow immunoassay NG-Test VanB (NG Biotech) was evaluated for the rapid detection of VanB-producing vancomycin-resistant enterococci (VanB-VREs) using 104 well-characterized enterococcal isolates. The sensitivity and specificity were both 100% when bacterial cells were grown in the presence of vancomycin used as a VanB inducer. The NG-Test VanB is an efficient, rapid and easy to implement assay in clinical microbiology laboratories for the confirmation of VanB-VREs from colonies. Together with the NG-Test VanA, they could replace the already existing tests available for the confirmation of acquired vancomycin resistance in enterococci, especially from selective media or from antibiograms, with 100% sensitivity and specificity. Rapid detection in less than 15 min will result in more efficient management of carriers and infected patients. In addition, these tests may be used for positive blood cultures, given a 3.5 h sub-culturing step on Chocolate agar PolyViteX in the presence of a 5-µg vancomycin disk, which is routinely performed in many clinical microbiology laboratories for every positive blood culture for subsequent MALDI-TOF identification of the growing bacteria.

## 1. Introduction

Vancomycin-resistant enterococci (VREs) are increasingly isolated worldwide and constitute a major public health concern [1,2,3,4]. Vancomycin resistance is due to the expression of van operons. Currently, there are eight different acquired vancomycin resistance operons described: vanA, vanB, vanD, vanE, vanG, vanL, vanM and vanN [4,5,6]. In Europe, the two main resistance phenotypes are by far VanA and VanB. Enterococci expressing VanA (VanA-VREs) display high levels of inducible resistance to both vancomycin and teicoplanin, whereas isolates expressing VanB (VanB-VREs) have variable levels of inducible resistance to vancomycin only [6,7].

The rapid detection of VREs is crucial to help implement appropriate infection control measures and to adapt antibiotic treatment for optimizing care strategies and outcomes [4,8]. Several culture-based methods, such as chromogenic or selective screening media, have been developed for VRE detection from rectal swabs. Detection can be optimized by inoculating clinical specimens in enrichment broth containing vancomycin followed by sub-culture on agar plates containing vancomycin [4,9]. These methods usually take 24–48 h, and as their specificity is low, colonies growing on these selective media require confirmatory testing, usually performed by PCR [8,9,10]. In addition, they may also lack sensitivity, especially with VanB-VREs with MICs of 4–8 µg/mL [4]. Molecular techniques are much faster, as compared to culture, as they may be used directly on rectal swabs, but the positive predictive value may also be low, especially for vanB genes that may be harbored by anaerobic bacteria as part of the intestinal microbiota, making culture mandatory to confirm every VanB-positive PCR result [10,11]. Similarly, the recently described vancomycin-variable *Enterococcus* (VVE) that bears the *vanA* gene is well detected using molecular methods, but has no phenotypic expression and does not grow on selective media [11,12]. The recently described NG-Test VanA lateral flow immunoassay (LFIA) was shown to be efficient for the detection of VanA-VREs from bacterial cultures [12].

Here, we have developed a rapid and reliable companion LFIA, the NG-Test VanB, for the detection of the second most prevalent VREs, i.e., VanB-VREs.

## 2. Materials and Methods

### 2.1. Ethics Statement and Monoclonal Antibodies

All animal experiments were performed in compliance with French and European regulations on the care of laboratory animals (European Community Directive 86/609, French Law 2001-486, 6 June 2001) and with the agreements of the Ethics Committee of the Commissariat à l’Energie Atomique (CEtEA) no. 12-026 and 15-055 delivered by the French Veterinary Services and CEA agreement D-91-272-106 from the Veterinary Inspection Department of Essonne (France). Ten-week-old Biozzi mice were immunized by intraperitoneal injection of purified recombinant VanB protein (50 µg), and the best pair of antibodies were produced on a large scale and provided to NG Biotech (Guipry, France) for the development of the NG-Test VanB assay, as previously described [12,13,14].

### 2.2. Cloning and Expression of the Recombinant VanB and Lysin plyV12 Protein in E. coli

The vanB gene of E. faecalis V583 (4) was PCR amplified using the primers VanB NdeI (5′-gatataCATATGaatagaataaaagttgcaatactg3′) and VanB XhoI (5′-gtggtgCTCGAGcccctttaacgctaatacgatcaa3′) and then cloned into the pET22b+ vector (Novagen; Merk, Darmstadt, Germany) [12,13,14]. Recombinant plasmids pET22b+ vanB and pET22b- plyV12 [12,15], which encodes a broadly active phage lytic enzyme with lethal activity against *E. faecalis* and *E. faecium*, were transformed into *Escherichia coli* BL 21 (DE3) (Novagen, Fontenay-sous-Bois, France). Upon induction, the recombinant VanB and plyV12 proteins were purified using Ni-NTA agarose affinity resin as previously described [12,13,14]. The VanB recombinant protein was then used to immunize mice and as a standard for the selection of monoclonal antibody (mAb) pairs as previously described [12,13,14].

### 2.3. Isolates Tested

The NG-Test VanB assay was validated using 135 well-characterized enterococci from the Associated French NRC for VREs (Hôpital Pontchaillou, Rennes, France) and from the Bicêtre Bacteriology laboratory isolate collection [12]. This panel included 84 VREs (33 VanB, 24 VanA, 8 VanC1, 12 VanC2, 3 VanD, 1 VanE, 1 VanG, 1 VanL and 1 VanN producers), 1 VanM-producing *E. coli* isolate, and 19 non-VRE isolates. Sixty-eight isolates were *E. faecium*, 20 *E. faecalis*, 8 *E. gallinarum* and 12 *E. casseliflavus* [12]. Two staphylococcal isolates (1 *Staphylococcus aureus* and 1 *Staphylococcus epidermidis*) and 2 carbapenemase-producing *Enterobacterales* (1 *E. coli* and 1 *Klebsiella pneumoniae*) were also included as negative controls.

### 2.4. Experimental Procedures on Colonies

The detection of VanB producers was first investigated using eight enterococcal isolates (3 VanB, 1 VanA, 1 VanC1, 1 VanC2 and 2 non-VRE) grown on different culture media widely used in routine: nine agar plates (Mueller–Hinton (MH), MH + a vancomycin disk of 5 µg, UriSelect^TM^ 4 and Bile Esculin Azide were from Bio-Rad (Marne-la-Coquette, France), ChromID^®^VRE, Columbia agar + 5% horse blood, Chocolate agar PolyViteX and D-Coccosel agar were from bioMérieux (Marcy-l-Etoile, France) and an in-house prepared MH agar plate supplemented with 6 µg/mL of vancomycin) and two broths (brain heart infusion (BHI, bioMérieux)) with and without a 30 µg vancomycin-containing disk (Bio-Rad).

For bacterial colonies, a 1-µL loop full of bacteria grown on the different agar plates was added to 100 µL extraction buffer (EB, provided by NG Biotech supplemented with 80 µg/mL of lysin (EB-80)). For bacterial broth culture, 500 µL culture was centrifuged in a table top Mikro 200R (Hettich, Sérézin du Rhône, France) for 5 min at 10,000 rpm (7000× *g*), and the pellet was resuspended in 100 µL of EB-80 as previously described [12,15]. After an incubation of 5 min at room temperature, the extract was loaded onto the cassette. The result was eye read after 15 min of migration by monitoring the appearance of a red band specific for VanB (test line, T), along with a band corresponding to the internal control (control line, C).

### 2.5. Experimental Procedures on Blood Cultures

Positive blood cultures with spiked enterococci (8 VanB-VREs, 7 VanA-VREs and 8 non-VanA and Van-B-VREs) were directly tested using 500 µL of positive blood culture processed using a modified broth protocol. The 500 µL positive blood culture was centrifuged for 5 min at 10,000 rpm (7000× *g*), and the pellet was resuspended in 500 µL of wash buffer, re-centrifugated and resuspended in 100 µL of EB-80 [12,15]. In addition, positive blood cultures were sub-cultured for 3.5 h on Chocolate agar PolyViteX plate (bioMérieux) with a 5 µg vancomycin-containing disk (Bio-Rad) Subsequently, colonies grown around the vancomycin disk were used for NG-TEST VanA [12] and NG-Test VanB assays, while other colonies were used for bacterial identification using the MALDI-TOF mass spectrometry identification system (Maldi Biotyper, Brucker Hamburg, Germany), as routinely performed in our laboratory. This process mainly consisted of the transfer of a thin layer of colonies onto a MALDI target (without transferring agar). Each sample was subsequently overlaid with 1 μL of 70% (*v*/*v*) formic acid and 1 μL of the matrix solution, which was dried at room temperature.

### 2.6. Detection Limit of the NG-Test VanB

The limit of detection (LOD) was determined in triplicate with two different VanB-VRE *E. faecium* isolates grown on MH or on ChromID^®^VRE agar plates (bioMérieux). McFarland bacterial suspension of 0.5 was serially diluted. A total of 100 µL of each dilution was mixed with 100 µL of EB containing 80 µg/mL of lysin (EB-80) and incubated for 5 min at RT prior to loading onto the cassette. Serial dilutions were also plated on MH plates to determine the exact cfu/mL.

## 3. Results

### 3.1. Performance of the NG-Test VanB on Different Culture Media

**Figure 1:**
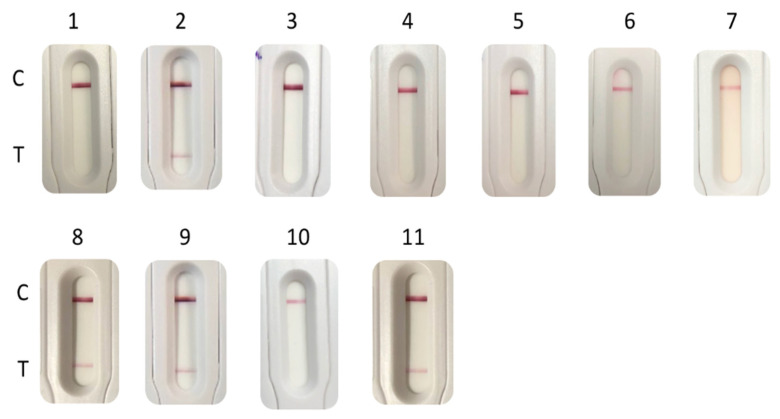
NG-Test VanB results obtained with 1-µL loop full of *E. faecium* VanB grown on different agar plates: (1) Müller–Hinton (MH); (2) MH with a 5-µg vancomycin disk; (3) Chocolate agar PolyViteX; (4) Columbia Agar + 5% horse blood; (5) UriSelect4; (6) Bile esculin agar; (7) D-Coccosel agar; (8) ChromID^®^VRE; (9) MH supplemented with 6 mg/L of vancomycin; and with 500 µL of overnight grown *E. faecium* VanB (10) in brain heart infusion (BHI); (11) and in BHI with a 30-µg disk of vancomycin. For 10 and 11, spun down bacterial pellets were resuspended in 100 µL of EB-80 and incubated for 5 min at RT prior to loading on the cassette. C stands for control line and T for test line.

The media tested in this study are those classically used to grow enterococci/VREs in many clinical bacteriology labs. All the tested enterococci grew on non-selective media (Müller–Hinton agar, Chocolate agar PolyViteX, Columbia Agar + 5% horse blood, UriSelect4 agar, Bile esculin agar, D-Coccosel agar and brain heart infusion liquid media), but in these media, the VanB determinant was not sufficiently expressed to be detected using the NG-Test VanB. With media containing vancomycin (MH with a 5 µg vancomycin disk, ChromID^®^VRE, and BHI + vanco), only VREs grew. The presence of vancomycin in these media allowed sufficient induction of the vanB operon to be detected by the NG-Test VanB (Table 1, Figure 1).

### 3.2. Performance of the NG-Test VanB and NG-Test VanA on VRE Screening Media

**Figure 2:**
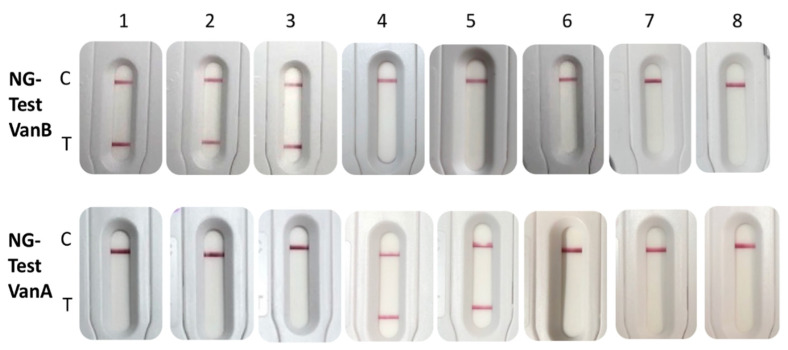
NG-Test VanB and VanA results obtained with 2-µL loop full of bacteria grown on ChromID^®^VRE, resuspended in 200 µL of EB-80 and incubated for 5 min at RT prior to loading 100 µL on each NG-Test VanB and NG-Test VanA cassette. The tested bacteria were (1) *E. faecalis* VanB; (2) *E. faecium* VanB; (3) *E. faecium* VanB; (4) *E. faecium* VanA isolate 12 (MIC Vancomycin/Teicoplanin 256/48 mg/L) [10]; (5) *E. faecium* VanA isolate 2 (MIC Vancomycin/Teicoplanin 16/6 mg/L) [10]; (6) *E. faecalis* ATCC29212; (7) *E. gallinarum* VanC1 BM4174; (8) *E. casseliflavus* VanC2 ATCC25788. C stands for control line and T for test line.

The NG-Test VanB and NG-Test VanA were tested using the same extract of bacteria grown on a commercially available vancomycin-containing medium used for VRE screening (ChromID^®^VRE, bioMérieux, France). Using this medium, both ligases were correctly detected (Figure 2).

The performance of the NG-Test VanB was further validated using a collection of 104 well-characterized enterococcal isolates grown on ChromID^®^ VRE, a medium classically used for VRE screening from stool samples [9] or on MH for non-VRE isolates.

All 33 VanB-VREs were detected in less than 15 min, while no-cross reaction was observed with other acquired determinants (i.e., VanA, C1, C2, D, E, G, L, M, N), non-VRE isolates and other species. These results showed that the NG-Test VanB had sensitivity and specificity both equal to 100% (data not shown).

### 3.3. Detection Limit

The limit of detection (LOD) could only be determined using VanB *E. faecium* isolates grown on ChromID^®^VRE agar plates (bioMérieux) that were subsequently serially diluted. The LOD was estimated at 0.95 +/− 0.2 × 10^7^ CFU per test. This LOD is two-log higher than that previously determined for the NG-Test VanA (4.9 10^5^ CFU/test) [12].

### 3.4. LFIA Results Directly from Blood Cultures

Detection directly from positive blood cultures using a previously described protocol [12] was not possible as VanB production was too low in the absence of induction by vancomycin. For VanA, as previously shown, this induction step was not necessary [12]. We therefore implemented an optimized protocol based on what is routinely performed in our laboratory for the identification of the bacteria present in positive blood cultures. Positive blood cultures are routinely plated on Chocolate agar PolyViteX plates and grown for 3.5 h at 37 °C under 5% CO_2_. The resulting bacterial lawn is then used to identify the bacteria by MALDI-TOF spectrometry. By adding a 5-µg vancomycin-containing disk to the plate, bacteria grown next to the disk could be tested using both NG-Test VanA and NG-Test VanB assays (Table 1).

**Figure 3:**
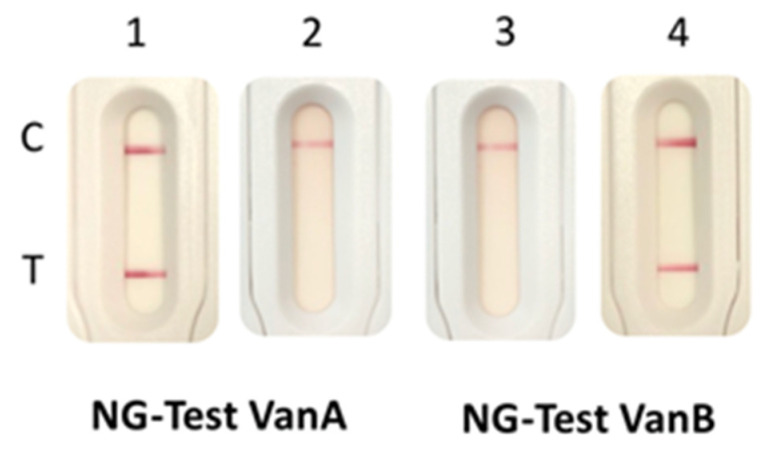
NG-Test VanB and NG-Test VanA results obtained with spiked blood cultures. Blood cultures were spiked with 10^4^ cfu of *E. faecium* VanA (1,3) and *E. faecium* VanB (2,4) and 10 mL of blood was incubated overnight. Subsequently, 100 µL of positive blood culture was plated on Chocolate agar PolyViteX (bioMérieux) and incubated for 3.5 h at 37 °C under 5% of CO_2_. A 2-µL loop full of bacteria grown next to a vancomycin disk was resuspended in 200 µL of EB-80 and incubated for 5 min at RT prior to loading on the NG-Test VanA (1,2) and NG-Test VanB (3,4) cassettes. C stands for control line and T for test line.

Using this protocol, *enterococci* were identified with MS scores > 1.7, thus allowing reliable identification at the species level. The presence of VanA or VanB could be evidenced using the two NG-Test strips, in less than 3 h and 45 min after blood culture was withdrawn from the automated incubator (BactAlert, bioMérieux) (Table 1, Figure 3). The presence of VanA and/or VanB could be evidenced at the same time the bacteria growing in the blood culture were identified.

## 4. Discussion

The accurate and rapid detection of VREs remains challenging and yet mandatory for infection control and for the treatment of infections caused by these bacteria. VanA and VanB are the most prevalent vancomycin-resistant determinants worldwide. The screening of VanA-VRE and VanB-VRE carriers may be performed by spreading rectal swabs on selective culture plates [9] or by molecular tools, which are faster but do not replace bacterial culture, especially with VanB-positive PCRs, as vanB genes may be present in anaerobic bacteria of the intestinal microbiota [8,9,10,11,12]. As selective culture media have low specificity, growing colonies need to be tested for the presence of vanA or vanB genes using generally molecular techniques [8,9].

**Figure 4:**
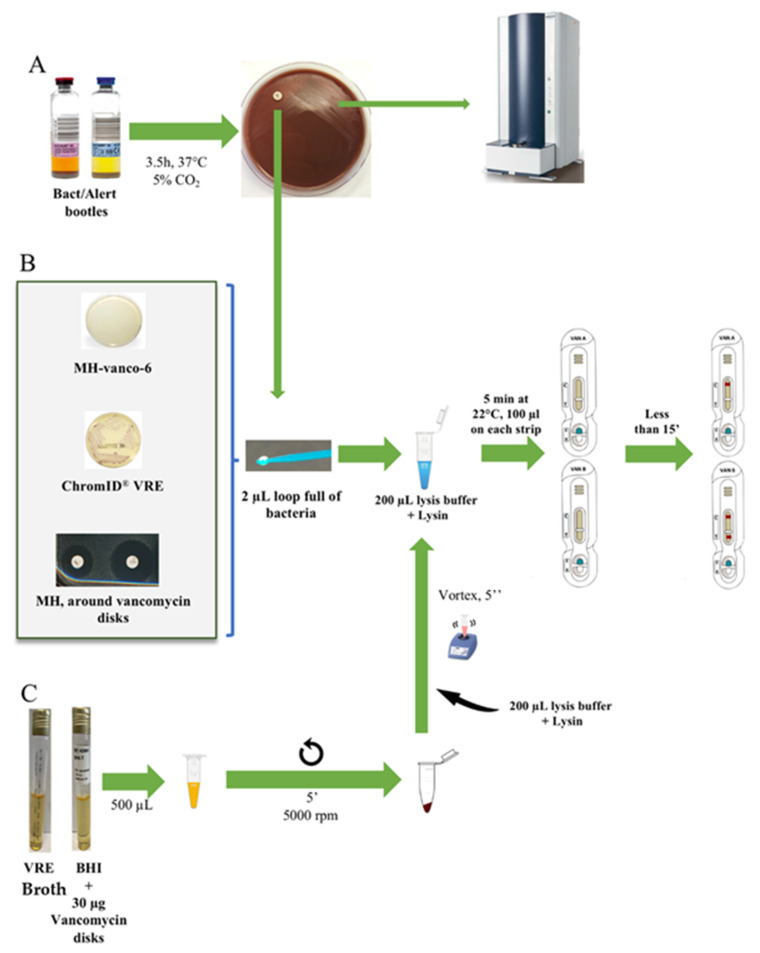
Use of NG-Test Van A and NG-Test Van B LFIAs. (A) NG-Test VanA and NG-Test VanB on spiked blood cultures. Blood cultures were spiked with 104 cfu of enterococci and 10 mL of blood was incubated overnight. Subsequently, 100 µL of positive blood culture was plated on Chocolate agar PolyViteX (bioMérieux) and incubated for 3.5 h at 37 °C under 5% of CO2. A 2-µL loop full of bacteria grown next to a vancomycin disk was resuspended in 200 µL of EB-80 and incubated for 5 min at RT prior to loading 100 µL on NG-Test VanA and 100 µL on NG-Test VanB cassettes. Bacterial lawn was also used for MALDI-TOF identification. (B) From o/n grown bacteria on agar plates. MH-Vanco-6: Müller–Hinton supplemented with 6 µg/mL of vancomycin; ChromID®VRE (bioMérieux); and MH with colonies grown next to a 5-µg vancomycin disk. A 2-µL loop full of bacteria was resuspended in 200 µL of EB-80 and incubated for 5 min at RT prior to loading 100 µL on NG-Test VanA and NG-Test VanB cassettes; and (C) from liquid broth. BHI: brain heart infusion and VRE broth were from bioMérieux. 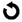 stands for centrifugation.

Here, we have developed a highly specific LFIA for VanB-VRE detection, with an easy and rapid extraction protocol suitable for routine use that could, together with the NG-Test VanA assay, complete/replace molecular confirmatory tests, either from colonies growing on selective VRE plates or from bacteria growing in vancomycin-containing enrichment broths (Figure 4). However, unlike the NG-Test VanA, the presence of vancomycin in the media, known to induce VanA or VanB ligase production through the activation of the sensor VanS [16], was mandatory to reliably detect VanB. The need for induction has previously been described for a VanA-LFIA that, even though it was specific, lacked sensitivity as it required overnight sub-culturing of the bacteria on vancomycin-containing Enterococcosel agar to induce VanA expression [17]. As compared to the NG-Test VanA that displayed a limit of detection of 6.3 × 10^6^ cfu and 4.9 × 10^5^ cfu per test with bacteria previously grown on MH and ChromID^®^ VRE plates (containing vancomycin), respectively, the LOD upon induction of NG-Test VanB was only 0.95 × 10^7^ cfu per test. This lack of sensitivity could either be due to the low basal level of Van B expression or to low affinities of anti-VanB antibodies used in the assay. The requirement of vancomycin (4–6 µg/mL) in order to induce VanB production could be perceived as a limitation of this assay. In fact, both NG-Test VanA and VanB assays will mainly be used as a confirmatory test for the presence of VREs, either from colonies growing on selective media containing vancomycin used for rectal screening for VRE carriage (such as ChromID VRE), from colonies growing next to the vancomycin-containing disk on a routine antibiogram of *enterococci* displaying reduced susceptibility to glycopeptides or from enrichment broth that is used to enrich rectal swabs with VREs (these media contain vancomycin). In all these situations, the presence of vancomycin in the media will allow the induction of VanB, and thus result in 100% detection of VanB using the NG-Test VanB.

For positive blood cultures, the lack of sensitivity of NG-Test VanB requiring a vancomycin induction step is clearly a drawback if direct detection from blood cultures is intended. However, a short induction with vancomycin may circumvent this limitation. In order to implement the NG-Test VanB in our routine workflow of positive blood cultures, a vancomycin disk (5 µg) was added to the 3.5 h subculture on Chocolate agar PolyViteX, which is routinely performed for every positive blood culture for subsequent MALDI-TOF mass spectrometry identification of the growing bacteria (Figure 4). Colonies grown next to the 5-µg vancomycin disk could thus be reliably identified as VanA or VanB-VREs. A 3 h incubation is the minimum time to observe a sufficiently grown bacterial lawn for MALDI-TOF analysis, but with 1/3 of the tests being inconclusive (score between 1.500 and 1.700). An additional 30 min of incubation improved bacterial identification to nearly 100% with a score >1.700. Our results are in agreement with a recent study that has shown that the short-term (5 h) subculture protocol was equivalent to the commercially available Sepsityper kit protocol (Brucker), with 85.2% and 64.3% of microorganisms correctly identified to the genus (score ≥ 1.700) and species levels (score ≥ 2.000), respectively. The short-term subculture revealed 89.6% and 70.4% of the correct identification of microorganisms to the genus and species levels, respectively, for 5 h before MALDI-TOF-MS analysis [18].

As blood stream infections with *E. faecalis* and *E. faecium* are relatively rare in France (4.6% and <1%, respectively, of the bacteria isolated during the national prevalence study of 2017 [19]) and vancomycin resistance is even rarer, with 0.1% in *E. feacalis* and 0.7% in *E. faecium* isolated from blood stream infections [20], the study was performed using spiked blood cultures and not clinical samples. Further prospective studies are thus necessary to estimate the performance of NG-Test VanA and VanB on real patients’ blood cultures using our protocol. In areas with high VRE prevalence, these assays might be used on every positive blood culture displaying chains of Gram-positive cocci, as revealed by Gram staining. In areas with low prevalence, it might be used on patients known to be VRE carriers and presenting chains of Gram-positive cocci in positive blood cultures or on patients hospitalized in a ward with an ongoing VRE outbreak.

## 5. Conclusions

The NG-Test VanB is easy to use, rapid and does not require any specific equipment or skills, while results are easy to read after 15 min of migration. As such, it can be easily implemented in the routine workflow of most clinical laboratories as a confirmatory test of VanB-VREs. Together with the NG-Test VanA [12], they could complete/replace the already existing panel of tests available for the confirmation of the most prevalent acquired vancomycin resistance in enterococci, especially from selective media or from enrichment broths (in the case of rectal screenings), or from antibiograms next to a vancomycin disk (in the case of infections), with a sensitivity and specificity both of 100% for the panel of isolates tested. They may also be used from positive blood cultures, given a 3.5 h sub-culturing step in the presence of a 5-µg vancomycin disk. This sub-culturing step is routinely performed in many clinical microbiology labs for bacterial identification using MALDI-TOF. Rapid detection in less than 15 min from colonies will result in more efficient management of carriers and infected patients. In the near future, the addition of emerging Van alleles, such as the VanD or VanM determinant, would allow the detection of most acquired resistance mechanisms encountered in VREs [4,21].

## Figures and Tables

**Table 1:** Results of the NG-Test VanB on colonies grown on different solid and liquid culture media and of the NG-Test VanB and NG-Test VanA for isolates from spiked blood cultures.

		Test Results on Various Culture Agar Plates Using NG-Test VanB ^a^									Test Results on Culture Broth Using NG-Test VanB ^b^				Test Results Using NG-Test VanA ^b^	
*van*-Gene Type	Isolates (*n* = Number of Tested Isolates)	MH	MH + Vanco Disk 5	Choc Agar	Blood Agar	Uri4	BAE	Cocco Agar	chromID^®^ VRE	MH Vanco-6	BHI	BHI + Vanco Disk 30	Positive Blood Culture	Blood Culture PVX (3.5 h) + Vanco Disk 5	Positive blood Culture	Blood Culture PVX (3.5 h) + Vanco Disk 5
*vanB*	*E. faecium* (*n* = 8)	−	+	−	−	−	−	−	+	+	VW ^c^	+	−	+	−	−
*vanA*	*E. faecalis* (*n* = 2) and *E. faecium* (*n* = 8)	−	−	−	−	−	−	−	−	−	−	−	−	−	+	+
*vanC1*	*E. gallinarum* (*n* = 4)	−	−	−	−	−	−	−	ng ^d^	−	−	ng	−	− ^e^	−	− ^e^
*vanC2*	*E. casseliflavus* (*n* = 6)	−	−	−	−	−	−	−	ng	ng	−	ng	−	− ^e^	−	−
Wildtype	*E. faecalis*	−	−	−	−	−	−	−	ng	ng	−	ng	−	− ^e^	−	−
Wildtype	*E. faecium*	−	−	−	−	−	−	−	ng	ng	−	ng	−	− ^e^	−	−

^a^ MH: Mueller–Hinton agar (Bio-Rad); MH + vanco disk 5: Mueller–Hinton agar + a vancomycin disk of 5 µg; Choc agar: Chocolate agar PolyViteX (bioMérieux); Blood agar: Columbia agar + 5% horse blood (bioMérieux); Uri4: UriSelect^TM^ 4 (Bio-Rad); BAE: Bile Esculin Azide Agar (Bio-Rad); Cocco agar: D Coccosel agar (bioMérieux); chromID VRE: chromID^®^ VRE (bioMérieux); MH-vanco-6: Mueller–Hinton agar supplemented with 6 µg/mL of vancomycin (Bio-Rad). Results were obtained using a 1-µL loop full of bacteria, resuspended in 100 µL of EB-80 and incubated for 5 min at RT prior to loading on the cassette. ^b^ BHI: brain heart infusion (bioMérieux); BHI + vanco disk 30: brain heart infusion + a disk of vancomycin of 30 µg (bioMérieux); BactAlert (bioMérieux) blood culture bottle spiked with 10^4^ cfu of enterococci and 10 mL of blood was incubated overnight. Five hundred µL of BHI or BHI vanco disk 30 culture was centrifuged for 5 min at 10,000 rpm (7000× *g*), the pellet was resuspended in 100 µL of EB-80 and incubated for 5 min at RT prior to loading on the cassette; Blood culture/PVX/3h: 100 µL of positive blood culture was plated on Chocolate agar PolyViteX (bioMérieux) and incubated for 3 h at 37 °C under 5% of CO_2_. A 2-µL loop full of bacteria grown next to a vancomycin disk was resuspended in 200 µL of EB-80 and incubated for 5 min at RT prior to loading on NG-Test VanA and NG-Test VanB cassettes. ^c^ VW: Very weak signal. ^d^: ng: no growth. ^e^ no growth next to the vancomycin disk, but colonies further away were used for LFIA assay.
